# Introduction to the Special Issue on Stem Cell and Biologic Scaffold Engineering

**DOI:** 10.3390/bioengineering6030072

**Published:** 2019-08-21

**Authors:** Panagiotis Mallis, Catherine Stavropoulos-Giokas, Efstathios Michalopoulos

**Affiliations:** Hellenic Cord Blood Bank, Biomedical Research Foundation Academy of Athens, 4 Soranou Ephessiou Street, Athens 115 27, Greece

**Keywords:** tissue engineering, regenerative medicine, stem cells, scaffolds, MSCs, iPSCs, nerve conduit, fibrin gel, scaffold classification, Wnt signaling

## Abstract

Tissue engineering and regenerative medicine is a rapidly evolving research field that effectively combines stem cells and biologic scaffolds in order to replace damaged tissues. Biologic scaffolds can be produced through the removal of resident cellular populations using several tissue engineering approaches, such as the decellularization method. In addition, tissue engineering requires the interaction of biologic scaffolds with cellular populations. Stem cells are characterized by unlimited cell division, self-renewal, and differentiation potential, distinguishing themselves as a frontline source for the repopulation of decellularized matrices and scaffolds. However, parameters such as stem cell number, in vitro cultivation conditions, and specific growth media composition need further evaluation. The ultimate goal is the development of “artificial” tissues similar to native ones, which is achieved by properly combining stem cells and biologic scaffolds, thus bringing artificial tissues one step closer to personalized medicine. In this special issue of *Bioengineering*, we highlight the beneficial effects of stem cells and scaffolds in the emerging field of tissue engineering. The current issue includes articles regarding the use of stem cells in tissue engineering approaches and the proper production of biologically based scaffolds like nerve conduit, esophageal scaffold, and fibrin gel.

Tissue engineering (TE) compromises an emerging field of the 21st century, where the repair or substitution of damaged tissues is still a clinical challenge. For the first time, in 1993, Langer and Vacanti proposed the definition of TE as “an interdisciplinary field that applies the principles of engineering and life sciences toward the development of biological substitutes that restore, maintain, or improve tissue function or a whole organ” [1]. For this purpose, TE could potentially be used in various regenerative medicine (RM) approaches, by efficiently combining stem cells and scaffolds. 

In this context, stem cells can be classified into embryonic and adult stem cells [2]. Embryonic stem cells (ESCs), which are referred to as pluripotent stem cells, can give rise to any cell type or tissue, and can be derived from early embryo stages, like blastocyst and inner cell mass [2]. Due to ethical concerns, the use of ESCs is limited in TE and RM approaches. Recently, a new type of pluripotent stem cells was generated in vitro by Takahasi and Yamanaka [3]. By introducing only four transcription factors, *Oct3/4, Sox2, c-Myc, and Klf4*, differentiated cells could erase their cell identity, through utilization of Polycomb and Trithorax group complexes, thus producing the induced pluripotent stem cells (iPSCs). However, the use of *c-Myc* in this process, a known oncogene, may significantly hamper the clinical application of iPSCs in TE and RM strategies. With this in mind, more research is needed to be performed in this field in order for the development of iPSCs to become clinically safe. On the other hand, mesenchymal stromal cells (MSCs), a mesodermal population that can be derived from both adult and embryonic tissues, may be used as an alternative cellular population for TE approaches [4]. According to the International Society for Cellular Therapies (ISCT), MSCs are non-hematopoietic plastic adherent cells, which can be differentiated into “chondrocytes”, “osteocytes”, and “adipocytes” [5]. Immunophenotypically, MSCs are expressing CD73, CD90, and CD105, while lacking expression of CD34, CD45, and HLA-DR [5]. MSCs can be derived from various sources, including Wharton’s jelly tissue, placental tissue, bone marrow, adipose tissue, dental pulp, liver, and lungs [4]. In addition, these cells can be easily expanded under in vitro culturing conditions for several passages without affecting their genome stability.

The field of TE relies on the use of various types of scaffolds, which can successfully mimic the biology of the extracellular matrix (ECM). Scaffolds provide a 3D microenvironment, where the cells can be adhered and proliferated under specific chemical and biophysical stimuli [6]. Furthermore, specialized bioreactor systems may contribute to the proper scaffold repopulation, cell proliferation and differentiation, even more. Scaffolds can be derived either from biological origin, including decellularized matrices, or can be fabricated in various dimensions, using mainly macromolecules derived from different origins, like expanded polytetrafluoroethylene (ePTFE), polyglycolic acid (PGA), polylactic acid (PLA), and polylactic co-glycolic acid (PLGA). The key properties of an ideal scaffold for TE can be summarized as (a) biocompatibility, (b) biodegradability, (c) mechanical properties, (d) easy fabrication, (e) non-toxic, and (f) proper cell attachment. Until now, scaffolds in combination with or without cells have been used in a wide variety of TE applications, including tendon and bone regeneration, blood vessel engineering, and trachea, heart, and esophagus development [7]. However, more research in this field must be performed by the scientific society in order to improve the clinical applications.

In this special issue of *Bioengineering*, we highlight the beneficial effects of stem cells and scaffolds in the emerging era of TE. The current issue included articles regarding the use of stem cells in TE and the proper production of biologically based scaffolds like nerve conduit, esophageal scaffold, and fibrin gel.

Under this scope, the immunoregulatory/immunosuppressive properties of MSCs that were derived from vitrified Wharton’s Jelly tissue are shown (specifically, MSCs expressed successfully the HLA-G, a non-classical HLA class I molecule, which is considered to be the main immunosuppressive agent during pregnancy) [8]. In this way, the MSCs could be administrated in injured sites, reducing the host immune response, thus contributing to tissue regeneration. The beneficial regenerative properties of MSCs have also been described in the pilot study of Protogerou et al. [9]. In this study, MSCs in combination with platelet lysate were administrated to treat patients with erectile disfunction (ED). The results showed the improvement of ED, which will be used for enrolling a wider study with a higher number of patients.

The use of iPSCs in TE and RM approaches might be very promising but still needs further clarification. Under this scope, Dias et al. [10] showed the dominant role of Wnt signaling in lineage commitment of human iPSCs. Moreover, the dominant effect of Wnt signaling over FGF and TGF-β was shown, resulting in the differentiation of iPSCs towards mesodermal lineages.

Regarding the biologically based scaffold development, Gontika et al. [11] described the utilization of decellularized human umbilical arteries (hUAs) as nerve conduits. Specifically, hUAs were obtained after gestation from umbilical cords and submitted to decellularization procedure. The produced scaffolds were free of cellular and nuclear material, while the ECM was preserved, as was observed by the histological analysis. Then, this scaffold was used as a nerve conduit in sciatic nerve injury. The results showed that the decellularized hUAs could support the elongation of nerve fibers and possibly could allow for the reinnervation of the target organs.

In a similar manner, the efficient development of a tissue engineered construct derived from rat esophagus was demonstrated [12]. In this study, rat esophagi were successfully decellularized. The ECM ultrastructure was retained after the decellularization procedure, and the obtained results could be used for scaling up this protocol to human tissues.

This special issue also included a preliminary study for fibrin gel production obtained from low volume cord blood units. The produced fibrin gel is characterized by several proteins that possibly contribute to tissue regeneration and possesses an alternative scaffold for wound healing.

Yuan et al. [13] introduce a new categorization method for scaffolds in order to avoid any misunderstandings between researchers. This new scaffold classification is of major importance, and is especially relevant in TE research.

The main scope of this special issue was to present state of the art tissue engineering approaches. Considerable effort has been undertaken by the scientific community toward the in vitro development of artificial organs such as heart, lungs, and liver [14]. The proper combination of stem cells and scaffolds under the conditions of good manufacturing practices (GMPs) could bring this form of personalized medicine one step closer to its clinical application. Finally, the editor would like to express his appreciation to the authors for their contribution to this special issue.
